# Role of body mass index in pregnancy outcomes after emergency cerclage for cervical insufficiency in singleton pregnant patients

**DOI:** 10.1186/s12884-023-05974-y

**Published:** 2023-09-07

**Authors:** Weiling Liu, Yaping Lu, Yuqin Fan, Guozhen Hei, Aijuan Zhang, Guoping Xue, Yanmei Wu

**Affiliations:** 1 Department of Obstetrics and Gynecology, Affiliated Maternity and Child Health Care Hospital of Nantong University, Nantong, 226007 China; 2 Department of Obstetrics and Gynecology, Shandong Provincial Maternal and Child Health Care Hospital Affiliated to Qingdao University, No. 238 Jingshi East Road, Jinan, 250014 China

**Keywords:** Obesity, Body mass index, Cervical insufficiency, Emergency cerclage, Amnioreduction, Maternal prognosis, Vaginal microecology

## Abstract

**Background:**

The study aims were to analyze pregnancy outcomes after the use of emergency cerclage in patients with different BMIs.

**Methods:**

A total of 76 singleton pregnant patients who underwent emergency cerclage at a tertiary comprehensive hospital in China between Jan 2017 and Dec 2021 were retrospectively divided into an obesity group of 37 patients with BMIs ≥ 28 kg/m^2^ and a non-obesity group of 39 patients with BMIs < 28 kg/m^2^. The medical records of patients were reviewed and all relevant clinical data were further collected into an itemized data spreadsheet for various analyses.

**Results:**

Emergent cerclage, along with amnioreduction if needed, could be safely performed on both obese and non-obese pregnant women with a dilated external cervix (> 1 cm), which effectively prolonged the gestational week up to ≥ 25 weeks. Obese gravidae had shorter suture-to-delivery intervals and mean pregnancy lengths but more spontaneous preterm births before 37 weeks, and a lower live birth rate (*P* < 0.05). Logistic regression analysis revealed that BMI, how many times cerclages have been performed during pregnancy (frequency of cerclage) and bacterial vaginosis, aerobic vaginitis and vulvovaginal candidiasis (vaginal microecology) were significantly correlated with fetal loss (*P* < 0.05), while rank correlation analysis established a negative correlation between BMI values and the suture-to-delivery interval (*P* = 0.031).

**Conclusions:**

Pregnant cervical insufficiency patients with BMIs > 28 kg/m^2^ may ill-serve the gestational outcomes and suture-to-delivery interval after their emergent cerclage. Additionally, BMI, frequency of cerclage and vaginal microecology accounted for higher fetal loss in patients who underwent emergency cerclage.

## Background

As one of the causes of premature birth, cervical insufficiency is responsible for about 8% of recurrent pregnancy losses in the second or third trimester [1], and for 0.2 – 7% of all complications encountered during pregnancy [2]. Therefore, more efforts are being made to diagnose and treat cervical insufficiency at an early stage.

Cervical cerclage is the most commonly used surgical approach to manage cervical insufficiency and has been included as the rescue operation in the American College of Obstetricians and Gynecologists (ACOG) guidelines [2, 3] with various indications. Emergency cerclage is usually performed in the second trimester on pregnant patients who present with a shortened and dilated cervix, sometimes even accompanied by amniocele. Its clinical significance, however, in salvaging pregnancy has not been unequivocally proven, especially when severe cervical insufficiency occurs. In addition, its related complications and adverse events have not been fully reported in the published literature. Therefore, it is not known whether the effects of emergency cerclage due to shortening or dilation of the cervix in the second trimester may potentiate the development of related complications [4].

Among all the possible contributory factors to premature birth due to cervical insufficiency, overweight and obesity became a clinical cynosure on their roles in devastating pregnant outcomes. A meta-analysis of more than one million pregnancies found that being both overweight and obese increased the risk of preterm birth before 32 gestational weeks [5]. Presumptively, being both overweight and obese may ill-prime pregnant women for various obesity-related gestational disorders. Although emergency cerclage has been used in clinical practice for dealing with cervical insufficiency -induced preterm births, few studies have looked into the possible link between the BMI status and the outcome of cerclage. However, whether being overweight or obese is associated with increased, decreased or neutral cerclage efficacy has largely remained unaddressed.

Thus, the present study aimed to evaluate the effectiveness of emergency cerclage in patients with different BMIs, explore the clinical value of emergency cerclage in improving pregnancy outcomes, and to analyze if a high BMI value was a significant risk factors affecting fetal loss.

## Methods

The present study retrospectively enrolled 76 singleton pregnant patients who had undergone emergency cerclage in the second trimester, between 14 and 28 weeks of pregnancy, at a tertiary hospital from Jan 2017 to Dec 2021. The inclusion criteria for the study were: patients found to have a dilated external cervix > 1 cm measured either by Doppler ultrasound or through vaginal examination; the amniotic sac in the cervical canal or vagina were revealed by vaginal peeping. The exclusion criteria were: active uterine contractions; clinical chorioamnionitis; vaginal bleeding; or life-incompatible fetal anomalies.

All the selected patients were further divided into either obesity or non-obesity groups according to their BMIs scores of more or less than 28 kg/m^2^, respectively. We assigned 28 kg/m^2^ as the cut-off value of BMI for the present study based on the cut-off value of 25.0 kg/m^2^ for the obesity of Asian people recommended by the International Obesity Task Force [6]. The medical records of patients were reviewed to extract all relevant clinical data: a history of pregnancy-associated diseases including gestational diabetes mellitus, autoimmune diseases, anemia and hypertensive disorders of pregnancy; and laboratory examination results, which were then databased into itemized spreadsheets for further analysis. Placentas were sent for pathological examination after delivery and the results retrieved from each patient’s medical record. Our study was conducted strictly following the Declaration of Helsinki guidelines. The study protocol (NO.2023-041) was reviewed and approved by the ethical institutions of Shandong Provincial Maternal and Child Health Care Hospital Affiliated to Qingdao University. The ethics committee agreed to waive the written informed consent due to the retrospective nature of the study.

Pregnancy-associated anemia was diagnosed for patients with hemoglobin concentrations < 110 g/L during pregnancy, postpartum hemorrhage with blood loss within 24 h after delivery of ≥ 500 mL, and very early preterm birth, with delivery < 28 weeks of gestational age in the present study. Chorioamnionitis was diagnosed by following the clinical guidelines issued by Queensland Health (2014) [7], while neonatal infection was diagnosed based on the sepsis 3.0 standard (2016) [8]. Based on the evidence of cardiorespiratory and neurological depression and evidence of acute hypoxic compromise with acidemia, the clinical diagnosis of perinatal asphyxia was an Apgar score > 7 as the parameter for a normal birth, and for a score < 7 and an arterial blood pH of < 7 for birth asphyxia [9].

The patients who did not present with preterm premature rupture of membranes (PROM) and met the criteria for emergent cerclage during the pre-operative evaluation underwent the procedure. Pre-operatively, the patients were given the prophylactic antibiotic ceftriaxone. Intra-operatively, the modified McDonald cervical cerclage procedures was performed, in which a purse-string stitch was placed to pinch the cervix closed tightly after the membrane was displaced from the operating field by a balloon inflated with 30 mL of normal saline solution under the sacculus within the endocervical canal to avoid inadvertent membrane puncture during suturing. Importantly, the post-cerclage length of the cervical canal should be > 2.0 cm.

During the procedure, if the amniotic cavity maintained a relatively high tension along with part of the amniotic sac protruding from the external os of the cervix after dilating the cervix, 100–150 mL of amniotic fluid was drained transperitoneally under ultrasonographical guidance with a 22G interventional aspiration needle to mitigate the difficulty of cerclage and to check possible amniotic abnormalities for further perioperative management. The drained amniotic fluid was sent for bacterial culture, a fluid smear and measurement of the glucose concentration, as well as interleukin-6 levels in order to evaluate the roles of intra-amniotic infection.

Perioperatively, the choice of antibiotic was justified by preoperative inflammatory indicators and reproductive tract infection indicators. Most cases were treated with prophylactic ceftriaxone, but cefoperazone-sulbactam could be administered if intra-amniotic infection was detected in the culture of aspirated amniotic fluid. Additionally, blood examinations and C reactive protein (CRP) every 3–7 days were retested to provide precise guidance for justifying the adjustment of the antibiotic therapy.

Vaginal and cervical secretion screening were performed around two weeks after the emergency cerclage. The cervical sutures were removed immediately if the patients showed any signs of PROM, ongoing infection, regular uterine contractions, inevitable abortion, premature delivery or had reached 36–37 weeks gestation.

### Statistical analysis

All data were analyzed using the SPSS version 19 (IBM, USA), and are expressed as means ± SD or frequencies. Either a χ^2^ test or Fisher’s exact probability method was conducted to compare count data, while Student’s *t*-test was employed to compare normally distributed measurement data. Logistic regression analyses were used to compare multiple factors and to calculate the odds ratios (ORs) at 95% confidence intervals (CIs) for maternal-infant outcomes. The Pearson rank correlation analysis was used to analyze any correlation between the BMI score and the suture-to-delivery interval. Missing data were processed by the list deletion method and the chain equation multiple interpolation methods. *P-*values < 0.05 were considered to be statistically significant.

## Results

### Demographics of patients

The clinical characteristics of the patients are presented in Table 1. Patients in the obesity group had significantly higher proportions of gestational diabetes mellitus, autoimmune diseases and hypertensive disorders of pregnancy, and a higher frequency of spontaneous preterm births (SPTB). Patients in the non-obesity group also had a significantly higher incidence of anemia. Moreover, all the other characteristics in the demographics, were not found to be significantly different between the two groups. It should be noted that none of the selected patients had a past history of cervical surgery.

**Table 1:** Demographics of patients in the non-obese versus obese group

Variables	Obesity group (*n* = 37)	Non-obesity group (*n* = 39)	*P*-value
Age (years), mean ± SD	31.95 ± 4.77	30.03 ± 3.57	0.050
Gravidity (freq), mean ± SD	2.51 ± 1.43	2.41 ± 0.97	0.711
Gestational weeks at cerclage (wk), mean ± SD	23.66 ± 2.11	22.66 ± 2.79	0.084
Nulliparous, *n* (%)	24 (64.9)	28 (71.8)	0.623
Prior 2nd trimester pregnancy loss, *n* (%)	15 (40.5)	9 (23.1)	0.139
Previous SPTB, *n* (%)	9 (24.3)	2 (5.1)	0.023
In vitro fertilization, *n* (%)	17 (45.9)	12 (30.8)	0.238
Cervical dilatation, *n* (%)			0.274
< 4 cm	31 (83.8)	28 (71.8)	
≥ 4 cm	6 (16.2)	11 (28.2)	
Frequency of cerclage, *n* (%)			0.303
Primary	31 (83.8)	36 (92.3)	
Secondary	6 (16.2)	3 (7.7)	
Pregnancy complications, *n* (%)			0.017
Gestational diabetes mellitus	6 (16.2)	4 (10.3)	
Autoimmune disease	2 (5.4)	0	
Anemia	7 (18.9)	20 (51.3)	
Hypertensive disorders of pregnancy	1 (2.7)	0	
Hospital stay (days), mean ± SD	19.70 ± 8.50	22.44 ± 5.45	0.098

Either a χ^2^ test or Fisher’s exact probability method was conducted to compare count data, student’s *t*-test was employed to compare normally distributed measurement data

Abbreviation: SPTB, spontaneous preterm births

### Comparison of laboratory indexes

The patients in the non-obesity group had a significantly lower incidence of vaginitis, including bacterial vaginosis (15.4% vs. 24.3%), aerobic vaginitis (12.8% vs. 32.4%) and vulvovaginal candidiasis (5.1% vs. 8.1%) than those in the obesity group. None of the patients in the study were found to have gonococcal and chlamydia infections. Sixteen patients (8 in each group) underwent amniotic fluid reduction before emergency cerclage, and only 2 patients in the non-obesity group had positive bacterial culture results after amniotic fluid analysis. In comparison to the laboratory test results, the pre-operative white blood cell (WBC) count and CRP were found to be significantly different in the obesity group compared to the non-obesity group. All remaining tests were not significantly different between the two cohorts of patients (Table 2).

**Table 2:** Comparison of laboratory indexes of patients in the non-obese versus the obese cohort

Variables	Obesity group (*n* = 37)	Non-obesity group (*n* = 39)	*P*-value
**Pre-operative**			
Albumin (g/L), mean ± SD	34.54 ± 2.65	33.95 ± 2.26	0.300
Glucose (mmol/L), mean ± SD	5.17 ± 0.94	5.01 ± 1.45	0.564
Hemoglobin (g/L), mean ± SD	114.38 ± 8.65	110.15 ± 12.99	0.101
WBC (× 10^9^/L), mean ± SD	10.52 ± 2.51	9.48 ± 1.98	0.049
Neutrophiles (%), mean ± SD	78.06 ± 5.18	78.05 ± 4.71	0.994
CRP (mg/L), mean ± SD	8.58 ± 5.06	6.06 ± 5.69	0.045
^**a**^ **Post-operative**			
Hemoglobin (g/L), mean ± SD	108.00 ± 11.07	105.14 ± 11.92	0.283
WBC (× 10^9^/L), mean ± SD	10.05 ± 3.33	10.08 ± 2.46	0.963
Neutrophiles (%), mean ± SD	77.81 ± 7.03	78.81 ± 6.47	0.523
CRP (mg/L), mean ± SD	22.39 ± 25.63	20.50 ± 22.88	0.735
Vaginal microecology			0.047
Bacterial vaginosis, *n* (%)	9 (24.3)	6 (15.4)	
Aerobic vaginitis, *n* (%)	12 (32.4)	5 (12.8)	
Vulvovaginal candidiasis, *n* (%)	3 (8.1)	2 (5.1)	
Mycoplasma, *n* (%)	16 (43.2)	14 (35.9)	0.513
^b^Amniotic fluid bacteria, *n* (%)	0	2 (25.0)	0.316

^a^Highest value of the postoperative examination

^b^Limited to patients with amniotic fluid reduction

Either a χ^2^ test or Fisher’s exact probability method was conducted to compare count data, student’s *t*-test was employed to compare normally distributed measurement data

Abbreviations: CRP, c-reactive protein; WBC, white blood cell

### Contributory factors to fetal loss

Since emergency cerclage was defined as a surgical failure in the present study, if the patient did not deliver a viable newborn, single factor analyses were performed to identify numerous factors that correlated with the fetal loss, including gestational weeks at emergency cerclage, BMI, frequency of cerclage, pre-operative WBC, post-operative WBC, post-operative CRP, vaginal microecology and mycoplasma. These factors subsequently underwent multivariate logistic regression analysis which revealed that BMI, frequency of cerclage and vaginal microecology were the main contributors to fetal loss after emergency cerclage (Table 3).

**Table 3:** Multiple factor logistic regression analysis of the risk factors for fetal loss

Variables	B	SE	Wald	DF	*P*-value	OR	95% CI of OR	
							Lower	Upper
BMI	-2.374	0.981	5.853	1	0.016	0.093	0.014	0.637
Frequency of cerclage	-3.632	1.673	4.711	1	0.030	0.026	0.001	0.703
Vaginal microecology	0.858	0.400	4.606	1	0.032	2.359	1.077	5.163

Logistic regression analyses were used to compare multiple factors and to calculate the ORs at 95% CIs for maternal-infant outcomes. Abbreviations: B, regression coefficient; CI, confidence interval; DF, degrees of freedom; OR, odds ratio; SE, standard error

The pregnancy outcomes of women who underwent emergency cerclage in the two cohorts are compared in Table 4. Both cerclage and amnioreduction did not produce any noticeable complications and the gestational time was extended for 8.99 ± 5.44 weeks after the suture operation. The suture-to-delivery interval and mean pregnancy length at delivery were significantly less but the rate of SPTB at < 37 weeks was significantly greater (81.2% vs. 59.0%; OR 2.981, 95% CI: 1.053 to 8.444) in the obesity group than in the other group (*P* < 0.05). However, the cumulative percentages of patients giving birth before 28-, 32- or 34-weeks’ gestation exhibited no obvious difference between the two cohorts. Similarly, our study did not find any significant differences in the incidence of PROM, postpartum hemorrhage and the frequency of caesarean sections. Chorioamnionitis occurred in 41 (53.9%) patients, with no significant difference between the two groups.

**Table 4:** Pregnancy outcomes of the patients with emergency cerclage in the non-obese versus obese cohort

Variables	Obesity group (*n* = 37)	Non-obesity group (*n* = 39)	*P*-value	OR (95% CI)
Suture-to-delivery interval (wk), mean ± SD	7.14 ± 4.80	10.75 ± 5.48	0.003	NA
Gestational age at delivery (wk), mean ± SD	30.72 ± 5.24	33.42 ± 5.35	0.028	NA
< 28 (wk), *n* (%)	12 (32.4)	7 (17.9)	0.145	0.456 (0.156–1.327)
< 32 (wk), *n* (%)	22 (59.5)	15 (38.5)	0.067	0.426 (0.170–1.070)
< 34 (wk), *n* (%)	24 (64.9)	17 (43.6)	0.063	0.419 (0.166–1.056)
< 37 (wk), *n* (%)	30 (81.2)	23 (59.0)	0.036	2.981 (1.053–8.444)
Cesarean delivery, *n* (%)	17 (45.9)	13 (33.3)	0.348	1.700 (0.672–4.300)
PROM, *n* (%)	19 (51.4)	19 (48.7)	0.818	1.111 (0.452–2.733)
Postpartum hemorrhage, *n* (%)	11 (29.7)	7 (17.9)	0.285	1.934 (0.657–5.694)
Chorioamnionitis, *n* (%)	20 (54.1)	21 (53.8)	0.985	1.008 (0.409–2.486)
Live birth, *n* (%)	23 (62.2)	33 (84.6)	0.026	0.299 (0.100–0.892)
Fetal distress, *n* (%)	21 (56.8)	12 (30.8)	0.022	0.339 (0.132–0.868)
NICU admission, *n* (%)	28 (75.7)	20 (51.3)	0.028	2.956 (1.110–7.866)
Neonatal infection, *n* (%)	18 (48.6)	9 (23.1)	0.020	3.158 (1.179–8.457)
^a^Birth weight (g)	2,426.5 ± 805.43	2,556.67 ± 899.22	0.581	NA

^a^Live births only

Either a χ^2^ test or Fisher’s exact probability method was conducted to compare count data and student’s *t*-test was employed to compare normally distributed measurement data between obesity group (*n* = 37) and non-obesity group (*n* = 39). Calculate the odds ratio (OR) and 95% confidence interval to assess the association and differences between two variables in two groups

Abbreviations: NA, not applicable; neonatal intensive care unit; PROM, premature rupture of membranes

All cases of live births contributed to delivery after 25 gestational weeks. The incidence of fetal distress (OR 0.339, 95%: 0.132–0.868) and neonatal intensive care unit (NICU) admission (OR 2.956, 95% CI: 1.110 to 7.866) in the obesity group was significantly higher than in the other cohort. Compared to the non-obesity group, the live birth rate was 62.2% in the obesity group, which was statistically lower (OR 0.299, 95% CI: 0.100 to 0.892), and the rate of neonantal infection development was significantly higher (OR 3.158, 95% CI: 1.179 to 8.457). No statistically significant differences were found in the birth weights between the two groups.

### Correlation analysis between the BMI value and the suture-to-delivery interval

Rank correlation analysis on the BMI score versus the suture-to-delivery interval found that the Pearson correlation coefficient (rs) was − 0.247 (*P* = 0.031, two-way), suggesting that the BMI score was negatively correlated with the suture-to-delivery interval.

## Discussion

Cervical insufficiency has significantly contributed to second-trimester pregnancy losses and preterm labor, but it can be managed with emergency cerclage on selected patients [10]. Since the clinical benefit of cerclage in gravidae with patients with different BMIs remains undefined in published reports, we conducted this retrospective cohort study on patients to investigate how emergent cerclage changes the pregnant outcomes and to identify which factors contributed to inferior outcomes of cerclage. The study demonstrated that emergent cerclage, along with amnioreduction if needed, could be safely performed on both obese and non-obese pregnant women with a dilated external cervix > 1 cm, and the cerclage sufficiently prolonged gestation up to 25 weeks and above. Thus, emergent cerclage can be chosen as the management option for cervical insufficiency.

Since being overweight and obese are communized in pregnant women nowadays, many studies have focused on the possible ill-effects of obesity on fetal development and pregnancy as well as delivery [11, 12]. However, some recent studies have tentatively overturned the prior putative claim on the contribution of obesity to preterm birth and cervical insufficiency. These authors purported that relative longer cervical s of obese gravidae could protect them against the development of preterm pregnancy and cervical insufficiency, thereby indicating that a longer cervix should speculatively lower the need for emergency cerclage as well as prolong the cerclage-to-delivery interval [13]. However, our study revealed that the length of pregnancy at delivery and the suture-to-delivery interval were significantly greater in the non-obesity group compared to the obese group and that a negative correlation existed between the BMI score and the suture-to-delivery interval based on a rank correlation analysis. Moreover, we further showed that obese gravidae had a shorter suture-to-delivery interval and mean pregnancy length but more SPTB at < 37 weeks, a lower live birth rate and a higher incidence of fetal distress, as well as NICU admission compared to the non-obese cohort (*P* < 0.05). Taken together, the present study suggested that a BMI score over 25 kg/m^2^ did produce an inferior outcome after cerclage.

Furthermore, multivariable analysis showed that live births numbers were correlated with BMI scores, the frequency of cerclage and vaginal microecology. One study [14] tentatively suggested a general adverse effect of higher BMIs on obstetrical outcomes and others reported that a second cerclage as well as abnormal vaginal microecology escalated the chances of chorioamnionitis, intrauterine infection and PROM, leading to potentially devastating pregnancy outcomes [15, 16]. It is noteworthy that Pang et al. [17] found the chance of chorioamnionitis occurring after emergency cerclage was 10%. In the present study, chorioamnionitis occurred in 53.9% of all patients, with no obvious difference between the two groups, a finding higher than previous research results, most likely due to the stricter standard of emergency cerclage. In addition, the role of amniocentesis in the treatment of cervical insufficiency has attracted more and more attention. Chalupska et al. [18] demonstrated that inflammation, being more frequent, was associated with worse outcomes in pregnancies with cervical insufficiency and prolapsed fetal membranes. In the present research, just 16 patients underwent amniotic fluid reduction and only 2 cases were positive for infection (12.5%). It was concluded, similarly, that most cases of intra-amniotic inflammation were not attributable to bacteria, but rather to sterile intra-amniotic inflammation. Thus, previous studies are more or less consistent with the results of our research on the risks of higher BMI scores, multiple cerclage and occult vaginitis for development during preterm pregnancy.

The levels of WBC and CRP have been practically accepted as indicators of the presence of pre-inflammatory processes. Significantly higher levels of pre-operative WBC and CRP were observed in our obese patients, indicating the possibility of overweight/obesity-induced inflammation in these pregnant women [19, 20]. Therefore, infection indicators can be considered as parameters and need to be strictly monitored before and after the cerclage operation. The use of antibiotics is justified during the perioperative period of emergent cerclage for better outcomes of the procedure and the associated pregnancy.

Studies that have been published on emergency cerclage for the management of urgent cervical insufficiency only considered time points for emergency cerclage between 22 and 28 weeks gestational, with the optimal cerclage-to-delivery interval being between 4 and 11 weeks [2]. In comparison, the present study did not find a remarkable difference from previous published research. Thus, the results may indirectly reflect the patients in our study who responded similarly to the effectiveness of emergency cerclage. Furthermore, they provided clear evidence that cerclage was a feasible and safe procedure to manage emergency and severe cervical insufficiency. Some retrospective studies have reported higher rates of procedure-related complications [16, 21], but no surgical complications occurred in our study. The management after emergency cervical cerclage also needs to pay much more attention to uterine contractions, with appropriate timely administration of uterine contraction inhibitors and timely removal of the cervical cerclage line to prevent postpartum hemorrhage caused by cervical laceration.

There were several limitations to the present study. First, since all our patients belonged to the ethnical category of Asian, for whom the International Obesity Task Force recommended lower cut-offs of BMI at 25.0 kg/m^2^ as the parameter of obesity, we chose a BMI of 28 kg/m^2^ as the divider between obese and non-obese patients in our study. As a result, the study finding should be cautiously applied to the Western gravidae, for whom a BMI ≥ 30 kg/m^2^ has been defined as obesity. Second, some relevant information was not retrieved from medical records during the retrospective review of the included patients. Third, many patients in this study who were transferred to our hospital had a strong need for emergency cerclage, so we did not add those patients to this retrospective study, which subsequently led to the analysis of data from limited numbers of patients. Fourth, it was a single-center study and lacked stringent requirements on the operating skills of obstetricians in their performance of cerclage, which might have increased the chance of bias in this study.

In conclusion, emergency cerclage can be safely performed in both obese and non-obese gravidae with a dilated external cervix, and the cerclage more effectively improves pregnancy outcomes with longer suture-to-delivery intervals and pregnancy length and better pregnant outcomes in the non-obese than obese gravidae. BMI scores, the frequency of cerclage and vaginal microecology are identified as risk factors that correlate with and predict the occurrence of cerclage failure.

## Data Availability

The datasets generated and analysed during the study are available from the corresponding author on reasonable request.

## Abbreviations

ACOG: American College of Obstetricians and Gynecologists
CRP: C reactive protein
GW: Gestational weeks
NICU: Neonatal intensive care unit
ORs: Odds ratios
PROM: Premature rupture of membranes
SPTB: Spontaneous preterm births
WBC: White blood cell
